# Sonic Hedgehog modulates EGFR dependent proliferation of neural stem cells during late mouse embryogenesis through EGFR transactivation

**DOI:** 10.3389/fncel.2013.00166

**Published:** 2013-09-26

**Authors:** Gisela Reinchisi, Margarita Parada, Pablo Lois, Claudia Oyanadel, Ronan Shaughnessy, Alfonso Gonzalez, Verónica Palma

**Affiliations:** ^1^Centro FONDAP de Regulación del Genoma, Facultad de Ciencias, Universidad de ChileSantiago, Chile; ^2^Departamento de Inmunología Clínica y Reumatología, Departamento de Biología Celular y Molecular, Facultad de Medicina, Facultad de Ciencias Biológicas, Centro de Envejecimiento y Regeneración, Pontificia Universidad Católica de ChileSantiago, Chile

**Keywords:** Shh, EGFR, Radial Glial Cells, transactivation, proliferation, cancer, neural progenitors

## Abstract

Sonic Hedgehog (Shh/GLI) and EGFR signaling pathways modulate Neural Stem Cell (NSC) proliferation. How these signals cooperate is therefore critical for understanding normal brain development and function. Here we report a novel acute effect of Shh signaling on EGFR function. We show that during late neocortex development, Shh mediates the activation of the ERK1/2 signaling pathway in Radial Glial cells (RGC) through EGFR transactivation. This process is dependent on metalloprotease activity and accounts for almost 50% of the EGFR-dependent mitogenic response of late NSCs. Furthermore, in HeLa cancer cells, a well-known model for studying the EGFR receptor function, Shh also induces cell proliferation involving EGFR activation, as reflected by EGFR internalization and ERK1/2 phosphorylation. These findings may have important implications for understanding the mechanisms that regulate NSC proliferation during neurogenesis and may lead to novel approaches to the treatment of tumors.

## Introduction

The mammalian neocortex develops from neural stem/progenitor cells (NSCs) that generate neurons, astrocytes and oligodendrocytes under the control of a complex array of environmental factors (Caviness et al., 1995). The maintenance of these cells plays a crucial role in normal brain development and homeostasis, and its misbehavior has been related to the origin of tumors (McCarthy, 2012). Among the molecules that regulate NSC proliferation and maintenance, Sonic Hedgehog (Shh) has been addressed as an important mitogenic factor that regulates NSC proliferation, both in the embryonic and adult brain, acting together with the Epidermal Growth Factor (EGF) (Caric et al., 2001; Palma and Ruiz i Altaba, 2004; Palma et al., 2005). Shh and EGF synergic action is necessary to induce late NSC proliferation and cross talk between the Shh and EGF signaling pathways has been reported based on canonical Shh/Gli activity and modulation of EGFR expression (Palma and Ruiz i Altaba, 2004; Bigelow et al., 2005; Palma et al., 2005; Ruiz i Altaba et al., 2007). Importantly, recent evidence has also involved Shh/Gli and EGFR cooperative interaction in oncogenic transformation (Schnidar et al., 2009). Therefore, a detailed understanding of the downstream processes and molecular players involved in this cooperative growth factor interaction are important not only for brain development but also for the identification of novel drug targets and rational-based combination therapies (Mimeault and Batra, 2010; Mangelberger et al., 2012).

Canonical Hedgehog signaling involves the binding of Shh to its receptor, Patched1 (Ptch1), relieving a repression of Ptch1 upon the co-receptor Smoothened (Smo), which is a GPCR presumably coupled to an inhibitory G-protein (Gαi) (Ogden et al., 2008; Lappano and Maggiolini, 2011). Activation of Smo decreases the production of cAMP via Gαi, thus PKA is inhibited and no longer phosphorylates the Gli transcription factors, the only well-known mediators of Shh, ultimately leading to their stabilization and activation (Huangfu and Anderson, 2006).

The EGF receptor of the tyrosine-kinase ERbB family (ERbB1-4) is one of the most widely distributed control systems of cell proliferation and differentiation, not only by responding to its own ligands but also serving as a nodal element for a variety of other stimuli (Carpenter, 1999; Yarden and Sliwkowski, 2001). Upon ligand binding, the receptor dimerizes and its intracellular tyrosine kinase domain becomes activated leading to phosphorylation of the receptor itself and several intracellular proteins with signaling or vesicular trafficking functions (Schlessinger, 2000; Sorkin and Goh, 2008). Activated EGFR, as other tyrosine-kinase receptors, signals via the Ras/Raf/MEK/MAPK, STAT, PI3K/AKT, and PLC-γ pathways and changes its broadcasting location during endocytosis and recycling, remaining active during variable periods of intracellular trafficking before entering the lysosomal-degradation route that finally ends its signaling activity (Schlessinger, 2000; Piper and Luzio, 2007; Sorkin and Goh, 2008).

It is well-known that besides its own stimulus, the EGFR can also be indirectly transactivated by signals emerging from a variety of other receptors, most notably by GPCRs coupled to Gαi or Gαq (Daub et al., 1996; Carpenter, 1999; Gschwind et al., 2001; Buvinic et al., 2007). Transactivation usually involves metalloprotease (MMPs)-mediated release of soluble EGFR ligands by cleavage of transmembrane ligand precursors at the cell surface (Izumi et al., 1998; Prenzel et al., 1999). Transactivation of EGFR has not been explored during late central nervous system (CNS) embryogenesis. It remains unknown whether EGFR function in NSCs is controlled by Shh via transactivation as recently described in embryonic stem cells (Heo et al., 2007). Here we studied the acute effect of Shh signaling over EGFR function in NSCs during late brain development. These cells are biologically characterized by the *in vitro* double requirement for Shh and EGF for cell proliferation. We show for the first time that Shh is capable of modulating EGFR-dependent proliferation of late cortex NSCs through EGFR mediated transactivation and endocytosis. We identified a subpopulation of NSCs constituted by Radial Glial Cells (RGC) as the main target of Shh. Moreover, we extended our results providing evidence that Shh also induced EGFR to mitogenic signaling, and to become endocytosed but not degraded, in HeLa cells, a well-characterized cancerous cell model for the study of EGFR function (Salazar and González, 2002; Buvinic et al., 2007; Sigismund et al., 2008; Norambuena et al., 2009). Thus, Shh can modulate EGFR signaling in different cell contexts. Such kind of control likely contributes to regulate the function of stem and progenitor cells during brain development and also the pathogenic arising and progression of several cancers.

## Materials and methods

### Reagents and antibodies

Cyclopamine (Infinity Pharmaceuticals, Inc.), recombinant octyl-modified Shh-N protein (R&D Systems), Purmorphamine (Calbiochem), EGF (human recombinant, Invitrogen), Tyrphostin (Calbiochem), Shh specific blocking antibody (5E-1), Shh-N plus the Gli inhibitor Gant61 (ALEXIS). Anti-phospho ERK, anti-total ERK, anti-β-actin and anti-β-tubulin, rabbit anti-GFAP, PD98059 were all from Sigma. Sheep anti-EGFR (Upstate), guinea pig polyclonal anti-GLAST, rabbit anti-Sox2, Ilomastat were from Chemicon, mouse anti-PKCλ (Transduction Labs), rabbit anti-caspase3 (Cell Signaling), polyclonal antibody EGFR984 (Biosonda Biotechnology), monoclonal antibody HB8506 (American Type Culture Collection), anti-phospho-tyrosine 4G10 monoclonal antibody (gift kindly provided by Dr. Maria Rosa Bono, Universidad de Chile, Santiago, Chile). Fluorescent secondary antibodies used were anti-rabbit Alexa488 and anti-mouse Alexa555 (Invitrogen).

### HeLa cell culture and treatments

An in-house population of HeLa cells previously characterized for EGFR internalization and transmodulation (Salazar and González, 2002; Buvinic et al., 2007; Norambuena et al., 2009) were cultured in DMEM supplemented with 10% FBS and antibiotics (100 U/ml penicillin and 100 μg/ml streptomycin), maintained at 37°C in a humidified atmosphere (95% air, 5% CO2). HeLa cells permanently expressing EGFR-GFP were obtained by transfection with pEGFP-N1-EGFR plasmid (kindly provided by Dr. Alexander Sorkin, University or Pittsburgh, USA) using the Lipofectamine 2000 method (Invitrogen). Selection was made in 1 mg/ml geneticin sulfate (G418) to obtain stable transfectants and the cells were then maintained in 0.8 mg/ml G418. Before the experiments, the cells were cultured to ~80% confluence and serum-starved for 24 h in media supplemented with 0.3% fetal bovine serum (FBS), unless otherwise indicated. Treatments were performed at 3.3 μg/ml recombinant Shh, hedgehog inhibitor Cyclopamine (Cyc) at 10 μM, Shh specific blocking antibody (5E-1) at 5 mg/ml, Gli inhibitor Gant61 at 10 μ M, Hedgehog agonist Purmorphamine (Pur) at 10 μ M and EGF at 1 and 50 ng/ml.

### HeLa cell RT-PCR and immunoblot

HeLa RNA preparation and RT-PCR specific reaction conditions and sequences for the human *hprt*,*ptc1*, and *gli1* primer pairs were as described (Palma and Ruiz i Altaba, 2004). For HeLa cells immunoblot assays, 60 mg protein from total cell extracts prepared as described (Salazar and González, 2002) were resolved on 10% polyacrylamide SDS gels and transferred onto nitrocellulose (Schleicher and Schuell, Germany). When required, EGFR was immunoprecipitated with the monoclonal antibody HB8506 and resolved by SDS-PAGE and immunoblotted with anti-ubiquitin P4D1 antibody, as described (Salazar and González, 2002). For total EGFR detection, membranes were stripped and incubated with the polyclonal antibody EGFR984 (Salazar and González, 2002). Immunoblots were revealed with ECL (Amersham Biosciences) and the bands were digitalized in a VISTA-T630 UMax scanner driven by Adobe Photoshop CS (Adobe Systems, Mountain View, CA).

### HeLa cell immunofluorescence and BrdU assay

To analyze EGFR internalization, HeLa EGFR-GFP stable cell clones were grown on glass coverslips, treated and fixed for 30 min at room temperature with 4% paraformaldehyde in PBS supplemented with 0.1 mM CaCl2 and 1 mM MgCl2 (PBS-CM). For BrdU incorporation assays, cells were grown in cover slips to ~50% confluence and serum-starved for 24 h in media supplemented with 0.3% FBS. Cells were treated with 3 mM of BrdU and BrdU detection was performed as previously described (Palma and Ruiz i Altaba, 2004) and marker-positive cells were assessed as percentage of DAPI-positive cells (5 random areas per experiment). All digital fluorescence images were acquired at the time indicated on a Zeiss Axiophot microscope with a Plan-APOCHROMAT 63X/1.4 oil immersion objective and the 14-bit Zeiss Axiocam camera.

### Neocortical explants and primary neurosphere culture and treatments

E18.5 cortical explants and Neurospheres (nsps) were obtained from outbred strains BalbC and C57/BL6 mice, respectively, and used for proliferation and differentiation and as described (Reynolds and Weiss, 1996; Dahmane et al., 2001), nsps for no more than three passages. Shh-N protein was used at 3.3 μg/ml. Other treatments included hedgehog inhibitor Cyclopamine (Cyc) at 10 μM, Hedgehog agonist Purmorphamine (Pur) at 10 μM and EGF 1 and 10 ng/ml. For acute stimulation of nsps Shh only was used. For proliferation assays nsps were plated on coated coverslips and cultured for 48 h in 10 ng/ml of EGF in the presence of Cyc or 1 ng/ml of EGF plus Shh (Palma and Ruiz i Altaba, 2004). To evaluate a possible role for the Shh pathway in RG cell maintenance nsps were deprived of EFG and cultured in the presence or absence of Shh alone to permit cell differentiation for 7 days, exchanging media every 3 days. Pharmacological inhibition with Cyc was performed similarly.

All animal procedures were in accordance with the Chilean legislation and were approved by Institutional Animal Care and Use Committees.

### Neurosphere BrdU incorporation and immunofluorescence

Incorporation of BrdU (3 mM, 2 h prior to culture fixation) and immunofluorescence detection on NSCs was performed as previously described (Dahmane et al., 2001) and marker-positive cells were assessed as percentage of DAPI-positive cells (5 random areas per experiment, from at least three independent experiments). Cells undergoing apoptosis were identified by caspase-3 immunodetection.

### Neurosphere immunoblot and immunoprecipitation

For immunoblot and immunoprecipitation, extracts of nsps or explants were prepared in lysis buffer (10mM Tris-Hcl, 5mM EDTA, 150 mM NaCl buffer and 1% Triton X-100) containing proteases and phosphatase inhibitors as described (Salazar and González, 2002). Proteins in immunoblots were visualized by ECL (Pierce) using horseradish peroxidase-conjugated secondary antibodies (Jackson Immuno Research).

### Flow cytometry on neocortical explants

Single cells dissociated from E18.5 neocortical explants prepared following protocol described in (Dahmane et al., 2001) were fixed in 2% paraformaldehyde for 15 min, permeabilized with 0.05% saponin and incubated for 30 min on ice in 1% PBS-BSA with anti-GFAP or anti-EGFR followed by secondary FITC-conjugated antibodies were analyzed in a flow cytometer (FACSort; BD Pharmingen) with CellQuest software. Statistical analysis.

Results were analyzed using Student's *t*-test and One-Way ANOVA. Values were expressed as mean ± the standard error of the mean (SEM). Significance was set as *p* < 0.05.

## Results

Shh and EGF synergic action has recently been involved in cell proliferation in embryonic stem cells (ES), and can occur via Shh induced EGFR transactivation (Heo et al., 2007), thus rising the question of how extensive this control system might be to other progenitor cell types or to other kind of cells, including cancerous cells. EGFR can be transactivated by a variety of stimuli. Depending on the kind and concentration of the ligand (e.g., EGF or TGF-a), the activated EGFR displays variations in signaling and cellular responses (Alwan et al., 2003; Sigismund et al., 2008; Madshus and Stang, 2009; Roepstorff et al., 2009). It is therefore important to analyze the contribution of Shh to the modulation of EGFR function according to cell type and context.

## Shh mediates NSC proliferation trough EGFR modulation

**Figure 1:**
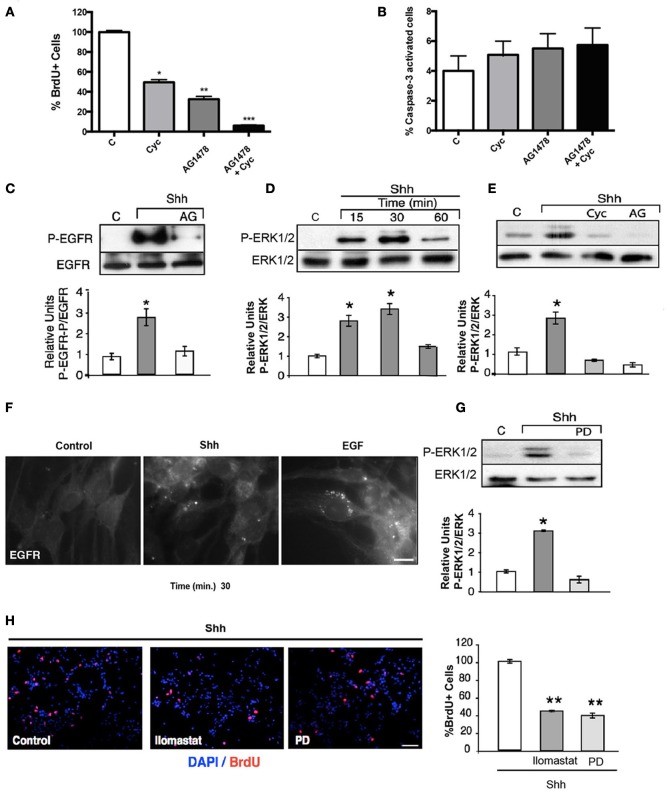
**Shh stimulation transactivates the EGFR leading to mitogenic signaling via the ERK1/2 pathway. (A)** EGF-dependent NSC proliferation requires the Shh signaling pathway. Quantification of the percentage of BrdU+ cells. The mitogenic activity decreased significantly with either cyc (50%; ^*^p < 0.03) or AG1478 (70%; ^**^p < 0.01) and was almost completely abrogated with both inhibitors (90%; ^***^p < 0.001). **(B)** Cells were treated with EGF (10 ng/ml), in the presence of either Cyc (10 μM), AG1478 (100 nM) or both drugs. Cells were fixed and cell death was evaluated by positive caspase-3 labeling. The histogram shows that treatment with inhibitors does not significantly change the percentage of caspase-3-activated cells. Data represent the results of three independent experiments. **(C)** Transactivation of the EGFR by Shh. Shh (3 μg/ml) induced an increment in the phospho-tyrosine activity of the EGFR, which was abolished by AG1478 (100 nM), added 30 min before. Tyrosine phosphorylation of EGFR was detected by immunoblot after immunoprecipitation. **(D,E)** Shh induces EGFR-dependent ERK1/2 activation. Nsps treated with Shh for the indicated time periods show increased phospho-ERK, detected by immunoblot, maximal at 30 min. Independent experiments show that such ERK1/2 activation measured after 15 min of Shh treatment is not only inhibited by Cyc (10 μM), but also by AG1478 (100 nM) (^*^p < 0.03). **(F)** Shh treatment in NSCs induces EGFR internalization. An adherent culture of nsps treated with 3.3 μg/ml Shh for 30 min shows EGFR internalization. EGF 50 ng/ml treatment was used as positive control. **(G)** MEK inhibitor PD98059 added 30 min before the stimulus inhibits Shh mediated ERK activation. (^*^p < 0.03). **(H)** Immunofluorescence for BrdU shows that ERK1/2 signaling and metalloprotease activity mediate Shh-dependent NSC proliferation. Quantification of the percentage of BrdU+ cells stimulated with Shh show a decreased proliferation of 60% by both PD98059 (100 μM) and the metalloprotease inhibitor Ilomastat (20 μM), added 30 min before stimulation. Data represent mean ± SEM of 3 independent experiments in triplicate (^**^p < 0.01). Bar = 10 μm **(F)** and Bar = 20 μm **(H)**.

During development, NSCs exhibit different requirements for growth factors. This is reflected in the growth of nsps, which require basic fibroblast growth factor (bFGF) at earlier embryonic stages and both Shh and EGF at late stages (Palma and Ruiz i Altaba, 2004). Considering that EGFR-mediated proliferation of cortical NSCs specifically requires EGF and Shh during late brain development, we first examined the effect of the specific inhibitors, Cyc (Hedgehog antagonist) and tyrphostin (EGFR inhibitor, AG1478) (Ward et al., 1994; Chen et al., 2002), on cell proliferation of E18.5 EGF-responsive nsps. Dissociated nsps were plated on substrate-coated dishes and grown in the presence of EGF (10 ng/ml) for 48 h. The cells were deprived of any growth factor for 2 h to cause cell growth arrest and maximal exposal of the EGFR to the cell surface, and were then stimulated with EGF for 2 h in the absence or presence of Cyc or AG1478, added 30 min before ligand addition. EGF-induced cell proliferation, measured by BrdU incorporation, was reduced by 70% with AG1478 (100 nM) and 50% with Cyc (10 μM) treatment, in agreement with previous reports confirming basal Shh activity (Dahmane et al., 2001; Palma and Ruiz i Altaba, 2004). Interestingly, simultaneous treatment with AG1478 and Cyc almost completely abrogated cell proliferation (about 96%) (Figure 1A), without significantly affecting apoptosis (Figure 1B). These results corroborate that the EGFR and Shh signaling pathways collaborate in the regulation of NSC proliferation during late embryogenesis. Interestingly, these observations also suggest that besides regulating the level of EGFR expression, Shh substantially contributes to the mitogenic response elicited by EGF signaling.

## NSC proliferation induced by Shh requires EGFR transactivation

The collaborative action of EGF and Shh can be exerted by the activation of independent signaling pathways that might converge upon yet unknown downstream elements. Alternatively, the EGFR might constitute an upstream element shared by both EGF and Shh. An obvious possibility is that Shh acutely transactivates the EGFR and as a consequence activates the Ras/Raf/MEK/ERK1/2 pathway involved in cell proliferation (Traverse et al., 1994; Seger and Krebs, 1995). We first studied the effects of Shh treatment over EGFR tyrosine-phosphorylation. Strikingly, the EGFR immunoprecipitated from nsps treated with Shh (3 μg/ml) for 15 min showed an increased phosphotyrosine content in immunoblots, which was abrogated by AG1478 (Figure 1C), thus reflecting EGFR activation. This Shh-induced EGFR activation led to ERK1/2 activation, detectable within 15 min and reaching a maximum by 30 min (Figure 1D), which was also abrogated by Cyc and AG1478 (Figure 1E). These results reveals for the first time that Shh transactivates the EGFR and its downstream Ras/Raf/MEK/ERK1/2 signaling pathway in cortical NSCs.

Because endocytosis plays a predominant role in EGFR function (Sorkin and Goh, 2008), and the intracellular trafficking and signaling consequences depend on the intensity of ligand-induced stimulation (Sigismund et al., 2008), we asked whether the transactivation elicited by Shh might led to receptor internalization. To answer this question we dissociated and plated nsps on substrate-coated dishes so as to obtain monolayers of cells that can be easily analyzed for EGFR endocytosis by indirect immunofluorescence. The cells were grown in the presence of EGF (10 ng/ml) for 48 h and depleted for 2 h before the experiments to induce maximal exposure of the EGFR to the cell surface. Treatment with Shh (3.3 μg/ml) induced a redistribution of EGFR from the cell surface to juxtanuclear endosomal compartments, although of less magnitude compared with EGF (50 ng/ml) used as positive control (Figure 1F). These results reveal that Shh transactivates the EGFR leading to its endocytosis.

In order to establish how much of the mitogenic effect of Shh is mediated by the EGFR-dependent ERK1/2 pathway we tested the effect of inhibiting its activating-kinase, MEK, with PD98059 (Alessi et al., 1995). PD98059 (100 μM) almost completely inhibited ERK1/2 activation (Figure 1G) and reduced the mitogenic activity of nsps grown in the presence of Shh by 60% (Figure 1H). Thus, the mitogenic effect of Shh upon NSCs seems to relay substantially on transactivation of EGFR and the subsequent engagement of the ERK1/2 pathway.

The mechanism of EGFR transactivation usually involves the proteolytic release of ligands from the cell surface through the activity of MMPs on ligand precursors (Izumi et al., 1998; Prenzel et al., 1999). Ilomastat/(GM6001), currently used as a general inhibitor of MMPs (Izumi et al., 1998; Prenzel et al., 1999), reduced Shh-dependent proliferation of nsps by 55% (Figure 1H), indicating that a similar MMP-dependent mechanism is triggered by Shh. These results indicate that the mitogenic signals conveyed by Shh acute stimulation requires transactivation of the EGFR through pathways involving metalloprotease mediated release of EGFR ligands from the cell surface.

## Radial Glial cells (RG) are the main targets of Shh signaling during late cortical development

**Figure 2:**
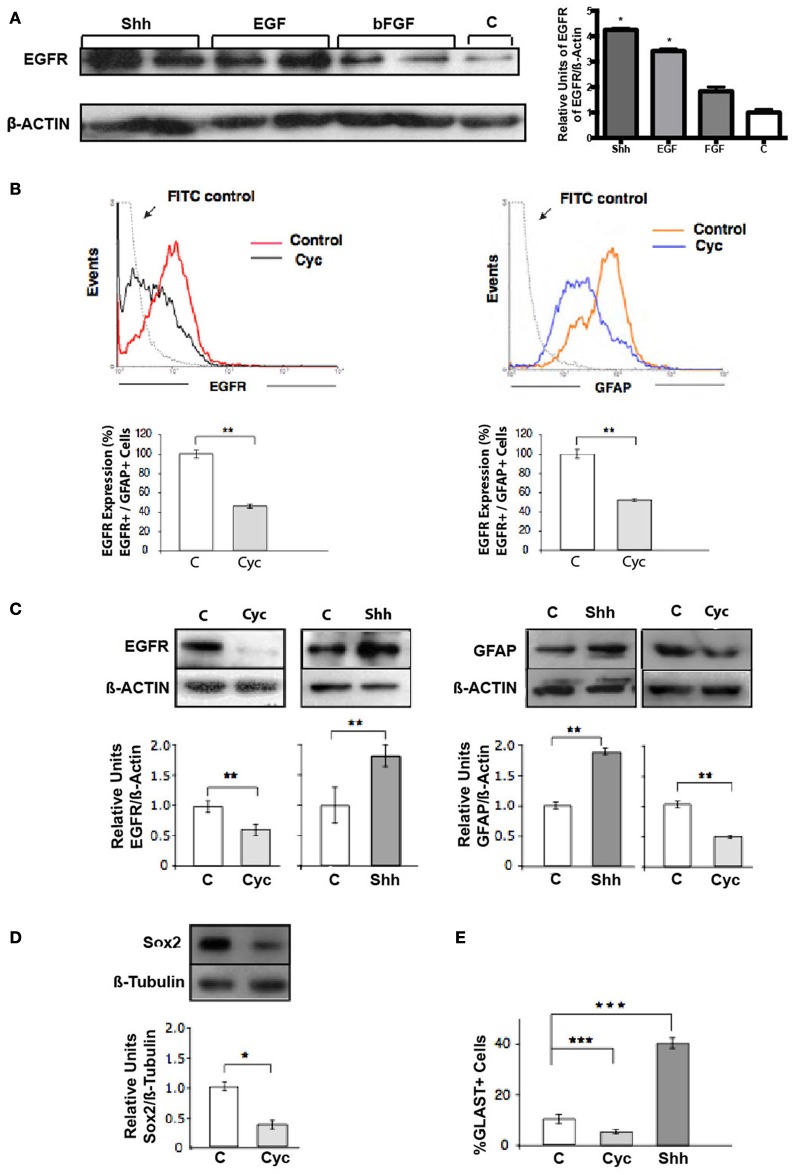
**Shh controls the pool of RG cells (GFAP+, Sox2+, GLAST+) that express EGFR. (A)** Western blot and densitometry analysis of EGFR show higher levels of EGFR in Shh-treated explants in comparison to samples treated with bFGF and positive control EGF. **(B)** Flow cytometry of cells harvested from E18.5 cortical explants. Treatment for 48 h with Cyc (10 μM) provokes similar decreases in the pool of EGFR- and GFAP-expressing cells. Histograms are representative of three independent experiments expressed as the percentage of the mean ± and SEM (^**^p < 0.01). **(C)** Western blot and densitometry analysis of EGFR of E18.5 nsps cultures treated for 48 h as indicated. **(D)** Immunoblots of Sox2 in NSCs. Treatment with Cyc for 48 h showed a decreased in Sox2 mass respect to β-tubulin. **(E)** Histograms showing the percentage of GLAST+ NSCs after 24 h treatment as indicated. Data represent the results of three independent experiments expressed as the percentage of the mean ± SEM (^*^p < 0.05, ^**^p < 0.01, ^***^p < 0.001).

Shh has been implicated in the upregulation of EGFR expression observed in NSC during late development, determining increased responsiveness to EGF. RT-PCR analysis of neocortical tissue has revealed that cyc treatment decreases the EGFR expression *in vivo* (Palma and Ruiz i Altaba, 2004). However, it has not been demonstrated that this modulation of EGFR expression translates to effective changes at the protein level. To test this hypothesis we compared the effect of Shh, EGF and bFGF on the expression of EGFR. EGFR expression increased significantly in E18.5 neocortical explants treated for 48 h with Shh only in comparison to bFGF or control samples, reaching even slightly higher levels when compared to a positive control treatment with EGF (Figure 2A). We next address the population of cells targeted by Shh in our model system. One candidate are the RG cells that during late states of embryonic development start to express GFAP (reviewed in Imura et al., 2003; Ihrie and Alvarez-Buylla, 2008) and some of them are EGF-responsive stem cells (Doetsch et al., 1999). We analyzed the expression of GFAP by flow cytometry on neocortical explants and found a significant decrement after 48 h of Cyc treatment (Figure 2B), similar to the reduction in EGFR expression. This observation suggests that Shh might be acting on GFAP+ cells that express EGFR. In addition, immunostaining of GFAP on 1 week plated single cell suspensions of nsps, showed that GFAP expression is significantly affected, decreasing by Cyc treatment and increasing by Shh only (Figure S1). Of note, we were able to distinguish more cells with the characteristic RG cell morphology after Shh treatment in comparison to control growth factor deprived cultures. GFAP is indeed a marker of NSCs both in the cortical region of late embryogenesis and in the subventricular zone (SVZ) of the adult, but it is also expressed in many terminally differentiated astrocytes. Other criteria is then needed to confirm that the RG-NSCs are the target of Shh. RG cells are characterized by Sox2 expression, which marks multipotent NSCs during different stages of mouse ontogeny (Graham et al., 2003; Ellis et al., 2004). The glutamate transporter (GLAST) is also a well-established marker of RG cells. Therefore, we assessed the effect of Cyc on the expression of these two markers. Nsp cultures cells grown in the presence of EGF (10 ng/ml) plus Cyc for 48 showed reduced levels of Sox2 by immunoblot (Figure 2D). Nsps treated with Cyc also reduced the number of cells expressing GLAST as evaluated by immunoblot and the opposite was seen upon treatment with Shh (Figure 2E). All these results strongly suggest that Shh acts on a specific progenitor pool: the RG cells.

Taken together, our studies establish for the first time that the stimulatory actions of EGF on NSC proliferation are mediated partially by the activation of the Shh signal transduction pathway.

## Shh acute stimuli modulate EGFR function in HeLa cancer cells

**Figure 3:**
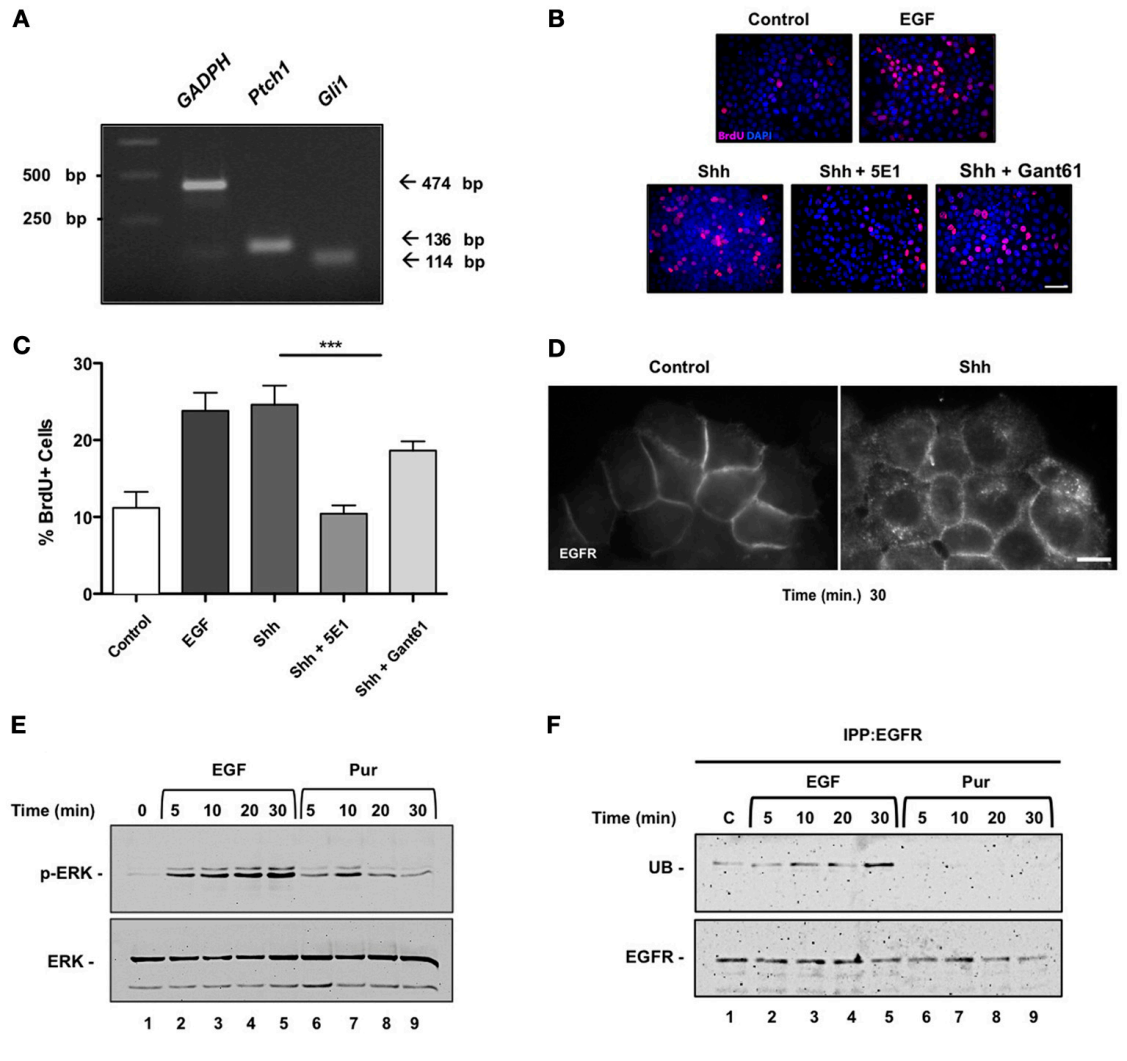
**Sonic Hedgehog induces internalization of EGFR, activation of ERK and proliferation of HeLa cells. (A)** Expression of Hedgehog signaling pathway components in HeLa cells. mRNA expression levels of *Gli1*, *Ptch1*, and *GAPDH* were assessed by reverse transcription-polymerase chain reaction (RT-PCR) in HeLa cells. (*M* = molecular mass; bp = base pair). **(B)** Shh induces proliferation of HeLa cells in a non-canonical fashion. HeLa cells were untreated (control) or treated with recombinant Shh or EGF as positive control, for 30 min before stimulation with Shh, Shh blocking antibody 5E1 (5E1.5 mg/ml) or Gli inhibitor Gant61 (Gant 10 μ M). Bar = 10 μm. **(C)** Quantification of the percentage of BrdU+ cells stimulated with EGF, Shh as indicated. A decrease in proliferation was observed in treatments with 5E1 and proliferation was not fully inhibited with Gant treatment. One-Way ANOVA *p* = 0.0002. **(D)** Shh treatment induces EGFR internalization. HeLa cells stably expressing EGFR-GFP were incubated in the absence (Control) or presence of Shh for 30 min and visualized under epifluorescence microscopy. Bar = 10 μm. **(E)** Both EGF (positive control) and Hedgehog agonist Pur acute treatments led to ERK1/2 signaling pathway activation in these cells. **(F)** Pur does not induce ubiquitination of EGFR. Cells were incubated in the absence (lane 1) or presence of either 50 ng/ml EGF (lanes 2–5) or 10 μ M Pur (lanes 6–9) for the indicated time periods. EGFR was then immunoprecipitated with mAb-HB8506, and its ubiquitin (UB) content was assessed by immunoblot with anti-ubiquitin mAb P4D1. Total mass of EGFR detected after incubating blots with polyclonal antibody EGFR984. In contrast to EGF, Pur did not induce ubiquitination of EGFR.

To assess whether Shh modulation of EGFR function is a more general phenomena that might be extensive to cancerous cells, we next performed similar experiments in HeLa cells. These cells have been widely used to study EGFR function (Salazar and González, 2002; Buvinic et al., 2007; Sigismund et al., 2008; Norambuena et al., 2009) but to our knowledge have not been studied as a Hedgehog signaling model system. Once ensured that HeLa cells expressed the components of the Hedgehog pathway through reverse transcriptase PCR (Figure 3A), we examined the effect of Shh stimulation on HeLa cell proliferation. Cells were deprived of any growth factor for 24 h and then incubated in absence (control) or presence of recombinant Shh, Shh plus its specific blocking antibody (5E-1), Shh plus the Gli inhibitor Gant61, or EGF as positive control for another 24 h (Figure 3B). Cell cycling, measured by BrdU incorporation after application of a 1 h pulse, showed that Shh induced cell proliferation of HeLa cells, which on the other hand showed a significant reduction in the presence of the Shh blocking peptide, 5E1. Interestingly, Shh treatment showed only a slight decrease in proliferation with the canonical pathway inhibitor Gant61 (Figure 3C). These results suggest that Shh might promote HeLa cell proliferation from a non-canonical source, without direct mediation of the Gli transcription factors.

One possibility is that Shh might be promoting cell proliferation by EGFR transactivation. Although we could not detect an increment of tyrosine-phosphorylation in EGFR assessed by immunoblot we did observe internalization of the receptor. HeLa cells, stably expressing EGFR-GFP, were depleted and incubated with or without Shh and EGFR internalization was examined under fluorescence microscopy after 30 min. Shh (3.3 μg/ml) induced a redistribution of EGFR from the cell surface to intracellular compartments indicative of EGFR endocytosis (Figure 3D). Both Shh and Hedgehog agonist Pur acute treatments led to ERK1/2 signaling pathway activation in these cells (Figures 2, 3E). Acute Pur treatment showed no evidence of EGFR degradation for up to 4 h (data not shown). Actually, as opposed to 50 ng/ml EGF treatment, we did not detect ubiquitination of the EGFR (Figure 3F). These results show that Shh drives EGFR internalization and promotes a transient mitogenic ERK1/2 signaling cascade activation. Shh seems to mimic the effects reported for low concentrations of EGF, which does not induce detectable ubiquitinylation and leads to EGFR recycling rather than degradation, thus contrasting with the effects of high ligand concentration (Sigismund et al., 2005, 2008, 2013). Actually, a threshold for EGFR ubiquitination has been recently reported in these cells (Sigismund et al., 2013). Overall, these results suggest that EGFR is also a downstream element in the Shh signaling pathway in HeLa cancer cells.

## Discussion

Acquisition and modulation of EGF responsiveness is critical in the regulation of several aspects of NSC development, such as self-renewal, differentiation and migration. Here we provide evidence that one of the main functions of Shh during late stages of brain embryogenesis is to promote and modulate EGF responsiveness of NSCs. We demonstrate that Shh has the capability to transactivate the EGFR and as a consequence strengthens the mitogenic intensity of the ERK1/2 signaling pathway triggered by EGF. This effect can explain the collaborative function of Shh and EGF exerted on the proliferation of late NSCs.

We identify RG (GFAP+, Sox2, and GLAST+) cells as the main target population of Shh, in which Shh induced transactivation, together with the reported EGFR expression (Dahmane et al., 2001) can all contribute to increase the EGF responsiveness. Our results are in line with a recent report by Dave et al., which showed *in vivo* that Shh pathway activation in the conditional Ptc1^Lox/Lox^; Nestin^Cre^ mutant cortex is mitogenic for cells that have a capacity to self-renew over an extended period of time. Authors identified RG cells- Nestin+/GLAST+ as the direct targets in the developing neocortex by mid neurogenesis (E14.5). Importantly, they showed reduction of Trb2 basal progenitor positive cells, suggesting that since RG cells can differentiate into basal progenitors, an indirect effect on this cell population could not be ruled out (Dave et al., 2011). In contrast Komada et al. reported that in *Shh*-CKO and *Smo*-conditional mutant embryos, the number of Tbr2-positive basal progenitor cells is significantly decreased in the SVZ/VZ of the developing neocortex (Komada et al., 2008). Furthermore, no significant differences could be observed in the Pax6 positive-RG cells among E15.5 wild-type and mutant mice. Clearly, this matter will require future investigation and we do not rule out that Shh signaling may be a mechanism for the regulation of both the number of RG cells and basal progenitors.

Our previous experiments in NSCs revealed synergism of the Shh and EGF signaling pathways reflected by increments of proliferative responses and induction of gene expression (Palma and Ruiz i Altaba, 2004). This up-regulation of EGFR expression levels induced by Shh indeed contributes to the collaborative action of Shh and EGFR on NSCs. EGFR expression has been associated with changes in progenitor cell behavior as a limiting factor for both proliferation and differentiation with response choice regulated in part by a concentration-dependent mechanism. Here we show that the mitogenic mechanism employed by Shh stimulus substantially depends on EGFR activity. Shh has been reported to provoke acute transactivation of EGFR, leading to cell proliferation in mouse embryonic stem cells (Heo et al., 2007). Our present results are the first to show that Shh transactivates the EGFR, leading to mitogenic ERK1/2 signaling in NSCs at late embryonic stages. Treatment of E18.5 cortical nsps with Shh for 15 min increases the tyrosine phosphorylation of the EGFR, a hallmark of its activation. Such Shh-induced EGFR phosphorylation was abrogated with an inhibitor of EGFR tyrosine kinase activity, and had functional consequences, as it resulted in activation of mitogenic ERK1/2 signaling. Recent observations suggest that the ability of cells to divide in response to EGFR activation requires the expression of a high level of the receptor in NSC (Lillien and Gulacsi, 2006). Because Shh can provoke an acute as well as a sustained status of EGFR activation (Bigelow et al., 2005; Heo et al., 2007), an initial event of EGFR transactivation can play a role in increasing the levels of the EGFR as well as in establishing regulatory loops acting on the proliferation machinery. Thus, Shh and EGF signaling pathways can functionally interact at different levels and may even establish a long-term regulatory loop depending on the cell context. How the synergistic signaling system of Shh and EGFR are integrated in NCSs and how does cooperativity between the pathways lead to selective activation of common response genes remains to be tested in future studies.

Endocytic trafficking is a crucial element in the regulation of EGFR function control (Goh and Sorkin, 2013; Pennock and Wang, 2003). The intensity, location and duration of receptor signaling can be modulated by the endocytic trafficking machinery, which is tightly intertwined with the mechanisms that control receptor signaling (Scita and Di Fiore, 2010; Goh and Sorkin, 2013). Activated EGFR undergoes transphosphorylation and ubiquitylation and these modifications define its endocytic trafficking/signaling behavior (Goh and Sorkin, 2013; Sigismund et al., 2013). Recent studies have shown that low ligand concentrations drive EGFR toward clathrin-dependent endocytosis followed by recycling, whereas high ligand concentrations promote EGFR internalization through a clathrin-independent route directed to lysosomal degradation (Sigismund et al., 2008). These alternatives have functional consequences in specifying the responses (Sigismund et al., 2008) and involve a threshold-controlled ubiquitylation system (Sigismund et al., 2013), which finely tunes the balance between EGFR signaling and trafficking. The ubiquitin-dependent down regulation route involves ESCRT-mediated sorting into intraluminal vesicles of multivesicular bodies that then fuse with lysosomes (Wegner et al., 2011). Thus, activated EGFR can remain signaling-competent for variable periods of time before degradation, determining different response outcomes (Sigismund et al., 2008; Rush et al., 2012). Our results, both in NSC and in HeLa cells show that Hedgehog pathway activation leads to EGFR internalization and to transient activation of the MAPK/ERK signaling cascade (Figure 1D, Figure S2), without detectable ubiquitination. This mimics the effect induced on EGFR by low EGF concentrations, which mainly leads to receptor recycling instead of degradation (Sigismund et al., 2008). In contrast, high EGF doses stimulation (50 ng/ml) induces EGFR ubiquitination and promotes its sorting to the lysosomal degradation pathway (Figure 3F). Besides mimicking EGFR stimulation by low EGF concentration, which by itself can constitute a strong mitogenic stimulus (Sigismund et al., 2008), another possibility of Shh-mediated regulation of EGFR function might occur through the reduction of PKA activity produced by activation of Smo via Gαi (Huangfu and Anderson, 2006). A decreased PKA activity has been shown to induce endocytosis and intracellular accumulation of empty/inactive EGFR, as well as an increment in the half-life of ligand-activated EGFR by delaying its sorting to the lysosomal degradation route (Salazar and González, 2002; Norambuena et al., 2010).

Our findings provide evidence that Shh, constitutes a mitogenic stimulating system, activating the EGFR and its MAPK/ERK signaling cascade. This potentiates the mitogenic effects of low EGF concentrations. Taking into account the recently described Shh constitutive autocrine modulation in NSCs (Bigelow et al., 2005; Ruiz i Altaba et al., 2007; Martínez et al., 2013), our results suggest that downstram activation of EGFR might provide the necessary strength to the intracellular pathways to mount a mitogenic response in NSCs. This might eventually increase the expression of EGFR, as previously reported for other EGFR transactivating receptors (Buvinic et al., 2007).

We suggest that during late development, neural precursor cell proliferation would be regulated by an interplay of mechanisms involving different signaling pathways, which would control the size of the progenitor pool. Additional approaches addressing the relationship of Shh, EGFR and others signaling pathways will be required order to obtain a better understanding of how, when and where these pathways interact to control NSC behavior.

Although at the moment specific mechanisms of EGFR transactivation by Shh are not defined, we show the intermediary action of MMPs in Shh-induced proliferation in NSCs. Our results show that Shh elicits a mitogenic response through activation of GM6001-sensitive MMPs. Inhibition of MMP activity attenuates the Shh-mediated cell proliferation indicating that the release of soluble ligands by induction of GM6001-sensitive MMPs after Shh stimulation could be responsible for cell proliferation. Our findings complement those described recently in ES cells by Heo et al. (2007). However, in NSCs, the identity of MMPs and their mechanism still remain to be defined.

Transactivation of the EGFR by agonist-activated GPCRs typically requires growth factor cleavage mediated by MMPs. It is noteworthy that the transactivation of the EGFR, mediated by members of the ADAM (a disintegrin and metalloproteinase) family of zinc-dependent proteases, is relevant in the development and progression of diverse types of human tumors. Furthermore, there is evidence suggesting that brain tumors resemble stem cell niches (Oliver and Wechsler-Reya, 2004). Brain tumors contain cancer stem cells, which are essential for both development and recurrence of the tumors (Dahmane et al., 2001; Vescovi et al., 2006). Together, these studies raise the possibility that the factors that regulate normal cell lineages from neural precursors may serve similar functions in the development of brain cancers from stem-like cancer cells (Ruiz i Altaba et al., 2007). Integration of the Shh and EGFR pathways could be a critical step in cancer initiation and/or tumor growth. Our data obtained in HeLa cells indeed suggest a conserved essential role of Shh on the regulation of EGFR function, which have therapeutic interest. The recognized importance of the EGFR in tumorigenesis suggests the possibility that abnormally increased Shh signaling contributes to carcinogenesis, thus providing additional rational to Shh as a target for antitumor therapies.

In conclusion, the present work gives further evidence of the importance of Shh in the regulation of the homeostasis of the EGF signaling pathways and proliferation, emphasizing cross-talk processes. Our findings shed new insight into the complex signal transduction pathways that mediate the actions of growth factors in the developing brain.

## Author contribution

Verónica Palma and Alfonso Gonzalez designed research; Gisela Reinchisi, Margarita Parada, Claudia Oyanadel, Ronan Shaughnessy, Verónica Palma, and Pablo Lois performed research; Gisela Reinchisi, Verónica Palma, and Pablo Lois analyzed data, Gisela Reinchisi, Margarita Parada, Alfonso Gonzalez, and Verónica Palma wrote the paper.

### Conflict of interest statement

The authors declare that the research was conducted in the absence of any commercial or financial relationships that could be construed as a potential conflict of interest.

## Supplementary material

Figure S1 **Shh maintains a specific GFAP+ -RG cell pool**. Nsps were cultured for 48 h with EGF (10 ng/ml) and then EGF was removed and the cells were cultured in the presence of only Shh (3 μg/ml) or Cyc (10 μM) for 7 days. RG phenotype on treated nsps was assessed by immunofluorescence staining for GFAP, counterstaining the cell nuclei with DAPI Bar = 50 μm. The percentage of cells expressing GFAP is depicted in the corresponding histogram. Shh treatment increases 2-fold the number of GFAP+ cells, whereas Cyc treatment shows the opposite effect, decreasing by 50% the number of GFAP+ cells. Values are the mean ± SEM of three experiments per conditions. (^**^*p* < 0.01 vs. control).

Figure S2 **Shh treatment in HeLa cells induces activation of the mitogenic ERK1/2 transduction pathway**. HeLa cells treated with 3.3 μg/ml Shh for the indicated time periods showed increased phospho-ERK, detected by immunoblot, maximal at 30 min.

## Abbreviations

NSC: neural stem cells
nsps: neurospheres
Shh: Sonic Hedgehog
CNS: Central Nervous System
Cyc: cyclopamine
Pur: purmorphamine.

## References

[B1] Alessi D. R. Cuenda A. Cohen P. Dudley D. T. Saltiel A. R. (1995). PD 098059 is a specific inhibitor of the activation of mitogen-activated protein kinase kinase *in vitro* and *in vivo*. J. Biol. Chem. 270, 27489–27494 10.1074/jbc.270.46.27489 7499206

[B2] Alwan H. A. J. van Zoelen E. J. J. van Leeuwen J. E. M. (2003). Ligand-induced lysosomal epidermal growth factor receptor (EGFR) degradation is preceded by proteasome-dependent EGFR de-ubiquitination. J. Biol. Chem. 278, 35781–35790 10.1074/jbc.M301326200 12829707

[B4] Bigelow R. L. H. Jen E. Y. Delehedde M. Chari N. S. McDonnell T. J. (2005). Sonic hedgehog induces epidermal growth factor dependent matrix infiltration in HaCaT keratinocytes. J. Invest. Dermatol. 124, 457–465 10.1111/j.0022-202X.2004.23590.x 15675968

[B5] Buvinic S. Bravo-Zehnder M. Boyer J. L. Huidobro-Toro J. P. González A. (2007). Nucleotide P2Y1 receptor regulates EGF receptor mitogenic signaling and expression in epithelial cells. J. Cell Sci. 120, 4289–4301 10.1242/jcs.03490 18057028

[B6] Caric D. Raphael H. Viti J. Feathers A. Wancio D. Lillien L. (2001). EGFRs mediate chemotactic migration in the developing telencephalon. Development 128, 4203–4216 11684657 10.1242/dev.128.21.4203

[B7] Carpenter G. (1999). Employment of the epidermal growth factor receptor in growth factor-independent signaling pathways. J. Cell Biol. 146, 697–702 10.1083/jcb.146.4.697 10459005 PMC2156131

[B8] Caviness V. S. Jr. Takahashi T. Nowakowski R. S. (1995). Numbers, time and neocortical neuronogenesis: a general developmental and evolutionary model. Trends Neurosci. 18, 379–383 10.1016/0166-2236(95)93933-O 7482802

[B9] Chen J. K. Taipale J. Cooper M. K. Beachy P. A. (2002). Inhibition of hedgehog signaling by direct binding of cyclopamine to smoothened. Genes Dev. 16, 2743–2748 10.1101/gad.1025302 12414725 PMC187469

[B10] Dahmane N. Sánchez P. Gitton Y. Palma V. Sun T. Beyna M. (2001). The Sonic Hedgehog-Gli pathway regulates dorsal brain growth and tumorigenesis. Development 128, 5201–5212 11748155 10.1242/dev.128.24.5201

[B11] Daub H. Weiss F. U. Wallasch C. Ullrich A. (1996). Role of transactivation of the EGF receptor in signalling by G-protein-coupled receptors. Nature 379, 557–560 10.1038/379557a0 8596637

[B12] Dave R. K. Ellis T. Toumpas M. C. Robson J. P. Julian E. Adolphe C. (2011). Sonic hedgehog and notch signaling can cooperate to regulate neurogenic divisions of neocortical progenitors. PLoS ONE 6:e14680 10.1371/journal.pone.0014680 21379383 PMC3040755

[B13] Doetsch F. Caillé I. Lim D. A. García-Verdugo J. M. Alvarez-Buylla A. (1999). Subventricular zone astrocytes are neural stem cells in the adult mammalian brain. Cell 97, 703–716 10.1016/S0092-8674(00)80783-7 10380923

[B14] Ellis P. Fagan B. M. Magness S. T. Hutton S. Taranova O. Hayashi S. (2004). SOX2, a persistent marker for multipotential neural stem cells derived from embryonic stem cells, the embryo or the adult. Dev. Neurosci. 26, 148–165 10.1159/000082134 15711057

[B15] Goh L. K. Sorkin A. (2013). Endocytosis of receptor tyrosine kinases. Cold Spring Harb. Perspect. Biol. 5:a017459 10.1101/cshperspect.a017459 23637288 PMC3632065

[B16] Graham V. Khudyakov J. Ellis P. Pevny L. (2003). SOX2 functions to maintain neural progenitor identity. Neuron 39, 749–765 10.1016/S0896-6273(03)00497-5 12948443

[B17] Gschwind A. Zwick E. Prenzel N. Leserer M. Ullrich A. (2001). Cell communication networks: epidermal growth factor receptor transactivation as the paradigm for interreceptor signal transmission. Oncogene 20, 1594–1600 10.1038/sj.onc.1204192 11313906

[B18] Heo J. S. Lee M. Y. Han H. J. (2007). Sonic hedgehog stimulates mouse embryonic stem cell proliferation by cooperation of Ca2+/protein kinase C and epidermal growth factor receptor as well as Gli1 activation. Stem Cells 25, 3069–3080 10.1634/stemcells.2007-0550 17901397

[B19] Huangfu D. Anderson K. V. (2006). Signaling from Smo to Ci/Gli: conservation and divergence of Hedgehog pathways from Drosophila to vertebrates. Development 133, 3–14 10.1242/dev.02169 16339192

[B20] Ihrie R. A. Alvarez-Buylla A. (2008). Cells in the astroglial lineage are neural stem cells. Cell Tissue Res. 331, 179–191 10.1007/s00441-007-0461-z 17786483

[B21] Imura T. Kornblum H. I. Sofroniew M. V. (2003). The predominant neural stem cell isolated from postnatal and adult forebrain but not early embryonic forebrain expresses GFAP. J. Neurosci. 23, 2824–2832 12684469 10.1523/JNEUROSCI.23-07-02824.2003 PMC6742109

[B22] Izumi Y. Hirata M. Hasuwa H. Iwamoto R. Umata T. Miyado K. (1998). A metalloprotease-disintegrin, MDC9/meltrin-gamma/ADAM9 and PKCdelta are involved in TPA-induced ectodomain shedding of membrane-anchored heparin-binding EGF-like growth factor. EMBO J. 17, 7260–7272 10.1093/emboj/17.24.7260 9857183 PMC1171072

[B23] Komada M. Saitsu H. Kinboshi M. Miura T. Shiota K. Ishibashi M. (2008). Hedgehog signaling is involved in development of the neocortex. Development 135, 2717–2727 10.1242/dev.015891 18614579

[B24] Lappano R. Maggiolini M. (2011). G protein-coupled receptors: novel targets for drug discovery in cancer. Nat. Rev. Drug Discov. 10, 47–60 10.1038/nrd3320 21193867

[B25] Lillien L. Gulacsi A. (2006). Environmental signals elicit multiple responses in dorsal telencephalic progenitors by threshold-dependent mechanisms. Cereb. Cortex 16 Suppl. 1, i74–i81 10.1093/cercor/bhj169 16766711

[B26] Madshus I. H. Stang E. (2009). Internalization and intracellular sorting of the EGF receptor: a model for understanding the mechanisms of receptor trafficking. J. Cell Sci. 122, 3433–3439 10.1242/jcs.050260 19759283

[B27] Mangelberger D. Kern D. Loipetzberger A. Eberl M. Aberger F. (2012). Cooperative Hedgehog-EGFR signaling. Front. Biosci. 17, 90–99 10.2741/3917 22201734 PMC3284771

[B28] Martínez C. Cornejo V. H. Lois P. Ellis T. Solis N. P. Wainwright B. J. (2013). Proliferation of murine midbrain neural stem cells depends upon an endogenous sonic hedgehog (Shh) source. PLoS ONE 8:e65818 10.1371/journal.pone.0065818 23776550 PMC3679138

[B29] McCarthy N. (2012). Cancer stem cells: tracing clones. Nat. Rev. Cancer 12, 579 10.1038/nrc3354 22898540

[B30] Mimeault M. Batra S. K. (2010). Frequent deregulations in the hedgehog signaling network and cross-talks with the epidermal growth factor receptor pathway involved in cancer progression and targeted therapies. Pharmacol. Rev. 62, 497–524 10.1124/pr.109.002329 20716670 PMC2964899

[B31] Norambuena A. Metz C. Jung J. E. Silva A. Otero C. Cancino J. (2010). Phosphatidic acid induces ligand-independent epidermal growth factor receptor endocytic traffic through PDE4 activation. Mol. Biol. Cell 21, 2916–2929 10.1091/mbc.E10-02-0167 20554760 PMC2921116

[B32] Norambuena A. Metz C. Vicuña L. Silva A. Pardo E. Oyanadel C. (2009). Galectin-8 induces apoptosis in Jurkat T cells by phosphatidic acid-mediated ERK1/2 activation supported by protein kinase A down-regulation. J. Biol. Chem. 284, 12670–12679 10.1074/jbc.M808949200 19276072 PMC2675996

[B33] Ogden S. K. Fei D. L. Schilling N. S. Ahmed Y. F. Hwa J. Robbins D. J. (2008). G protein Galphai functions immediately downstream of Smoothened in Hedgehog signalling. Nature 456, 967–970 10.1038/nature07459 18987629 PMC2744466

[B34] Oliver T. G. Wechsler-Reya R. J. (2004). Getting at the root and stem of brain tumors. Neuron 42, 885–888 10.1016/j.neuron.2004.06.011 15207233

[B35] Palma V. Lim D. A. Dahmane N. Sánchez P. Brionne T. C. Herzberg C. D. (2005). Sonic hedgehog controls stem cell behavior in the postnatal and adult brain. Development 132, 335–344 10.1242/dev.01567 15604099 PMC1431583

[B36] Palma V. Ruiz i Altaba A. (2004). Hedgehog-GLI signaling regulates the behavior of cells with stem cell properties in the developing neocortex. Development 131, 337–345 10.1242/dev.00930 14681189

[B37] Pennock S. Wang Z. (2003). Stimulation of cell proliferation by endosomal epidermal growth factor receptor as revealed through two distinct phases of signaling. Mol. Cell. Biol. 23, 5803–5815 10.1128/MCB.23.16.5803-5815.2003 12897150 PMC166318

[B38] Piper R. C. Luzio J. P. (2007). Ubiquitin-dependent sorting of integral membrane proteins for degradation in lysosomes. Curr. Opin. Cell Biol. 19, 459–465 10.1016/j.ceb.2007.07.002 17689064 PMC2046217

[B39] Prenzel N. Zwick E. Daub H. Leserer M. Abraham R. Wallasch C. (1999). EGF receptor transactivation by G-protein-coupled receptors requires metalloproteinase cleavage of proHB-EGF. Nature 402, 884–888 10.1038/47260 10622253

[B40] Reynolds B. A. Weiss S. (1996). Clonal and population analyses demonstrate that an EGF-responsive mammalian embryonic CNS precursor is a stem cell. Dev. Biol. 175, 1–13 10.1006/dbio.1996.0090 8608856

[B41] Roepstorff K. Grandal M. V. Henriksen L. Knudsen S. L. J. Lerdrup M. Grøvdal L. (2009). Differential effects of EGFR ligands on endocytic sorting of the receptor. Traffic 10, 1115–1127 10.1111/j.1600-0854.2009.00943.x 19531065 PMC2723868

[B42] Ruiz i Altaba A. Mas C. Stecca B. (2007). The Gli code: an information nexus regulating cell fate, stemness and cancer. Trends Cell Biol. 17, 438–447 10.1016/j.tcb.2007.06.007 17845852 PMC2601665

[B43] Rush J. S. Quinalty L. M. Engelman L. Sherry D. M. Ceresa B. P. (2012). Endosomal accumulation of the activated epidermal growth factor receptor (EGFR) induces apoptosis. J. Biol. Chem. 287, 712–722 10.1074/jbc.M111.294470 22102283 PMC3249126

[B44] Salazar G. González A. (2002). Novel mechanism for regulation of epidermal growth factor receptor endocytosis revealed by protein kinase A inhibition. Mol. Biol. Cell 13, 1677–1693 10.1091/mbc.01-08-0403 12006662 PMC111136

[B45] Schlessinger J. (2000). Cell signaling by receptor tyrosine kinases. Cell 103, 211–225 10.1016/S0092-8674(00)00114-8 11057895

[B46] Schnidar H. Eberl M. Klingler S. Mangelberger D. Kasper M. Hauser-Kronberger C. (2009). Epidermal growth factor receptor signaling synergizes with Hedgehog/GLI in oncogenic transformation via activation of the MEK/ERK/JUN pathway. Cancer Res. 69, 1284–1292 10.1158/0008-5472.CAN-08-2331 19190345 PMC3035872

[B47] Scita G. Di Fiore P. P. (2010). The endocytic matrix. Nature 463, 464–473 10.1038/nature08910 20110990

[B48] Seger R. Krebs E. G. (1995). The MAPK signaling cascade. FASEB J. 9, 726–735 7601337

[B49] Sigismund S. Algisi V. Nappo G. Conte A. Pascolutti R. Cuomo A. (2013). Threshold-controlled ubiquitination of the EGFR directs receptor fate. EMBO J. 32, 2140–2157 10.1038/emboj.2013.149 23799367 PMC3730230

[B50] Sigismund S. Argenzio E. Tosoni D. Cavallaro E. Polo S. Di Fiore P. P. (2008). Clathrin-mediated internalization is essential for sustained EGFR signaling but dispensable for degradation. Dev. Cell 15, 209–219 10.1016/j.devcel.2008.06.012 18694561

[B51] Sigismund S. Woelk T. Puri C. Maspero E. Tacchetti C. Transidico P. (2005). Clathrin-independent endocytosis of ubiquitinated cargos. Proc. Natl. Acad. Sci. U.S.A. 102, 2760–2765 10.1073/pnas.0409817102 15701692 PMC549482

[B52] Sorkin A. Goh L. K. (2008). Endocytosis and intracellular trafficking of ErbBs. Exp. Cell Res. 314, 3093–3106 10.1016/j.yexcr.2008.08.013 18793634 PMC2605728

[B54] Traverse S. Seedorf K. Paterson H. Marshall C. J. Cohen P. Ullrich A. (1994). EGF triggers neuronal differentiation of PC12 cells that overexpress the EGF receptor. Curr. Biol. 4, 694–701 10.1016/S0960-9822(00)00154-8 7953555

[B55] Vescovi A. L. Galli R. Reynolds B. A. (2006). Brain tumour stem cells. Nat. Rev. Cancer 6, 425–436 10.1038/nrc1889 16723989

[B56] Ward W. H. Cook P. N. Slater A. M. Davies D. H. Holdgate G. A. Green L. R. (1994). Epidermal growth factor receptor tyrosine kinase. Investigation of catalytic mechanism, structure-based searching and discovery of a potent inhibitor. Biochem. Pharmacol. 48, 659–666 10.1016/0006-2952(94)90042-6 8080438

[B57] Wegner C. S. Rodahl L. M. W. Stenmark H. (2011). ESCRT proteins and cell signalling. Traffic 12, 1291–1297 10.1111/j.1600-0854.2011.01210.x 21518165

[B58] Yarden Y. Sliwkowski M. X. (2001). Untangling the ErbB signalling network. Nat. Rev. Mol. Cell Biol. 2, 127–137 10.1038/35052073 11252954

