# Supplementary figures and images for: Sonic Hedgehog modulates EGFR dependent proliferation of neural stem cells during late mouse embryogenesis through EGFR transactivation
Source: Front Cell Neurosci. 2013 Sep 26;7:166. doi: 10.3389/fncel.2013.00166 (PMC3783837; doi:10.3389/fncel.2013.00166)

Control

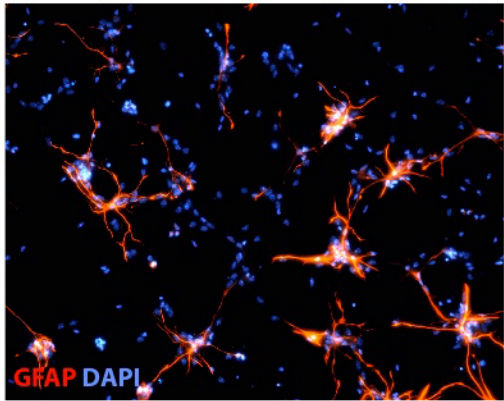

Cyc

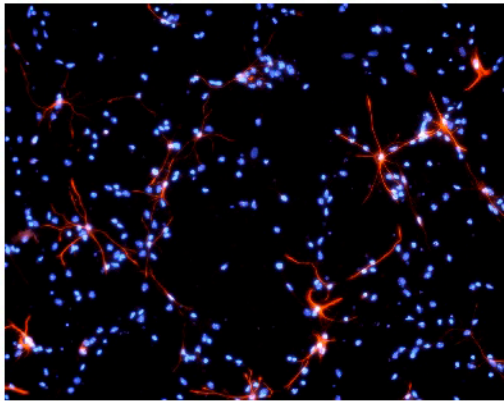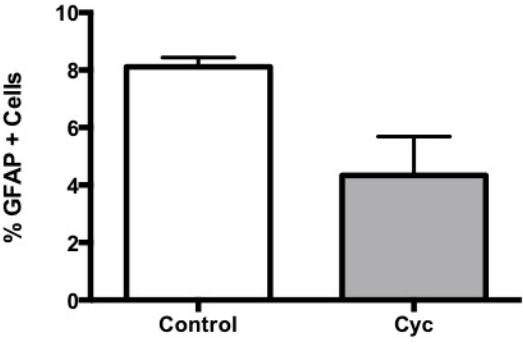

Control

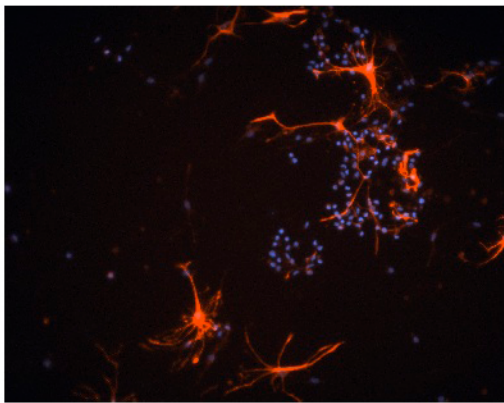

Shh

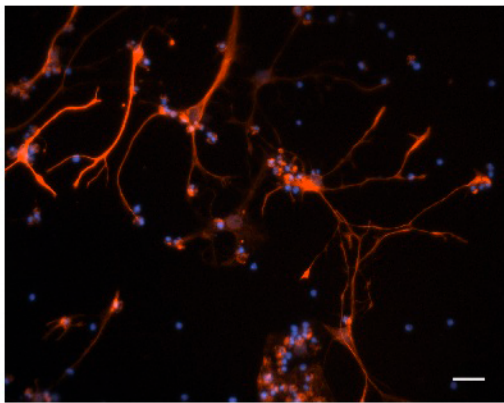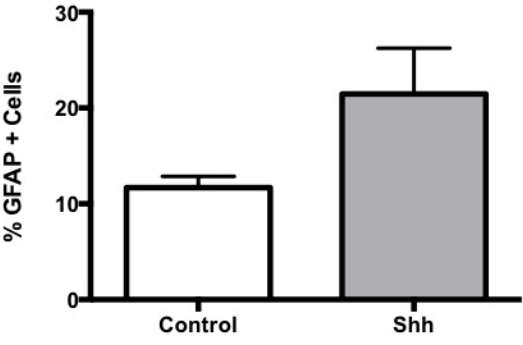

Supplement: Figure S1 — Shh maintains a specific GFAP+ -RG cell pool. Nsps were cultured for 48 h with EGF (10 ng/ml) and then EGF was removed and the cells were cultured in the presence of only Shh (3 μg/ml) or Cyc (10 μM) for 7 days. RG phenotype on treated nsps was assessed by immunofluorescence staining for GFAP, counterstaining the cell nuclei with DAPI Bar = 50 μm. The percentage of cells expressing GFAP is depicted in the corresponding histogram. Shh treatment increases 2-fold the number of GFAP+ cells, whereas Cyc treatment shows the opposite effect, decreasing by 50% the number of GFAP+ cells. Values are the mean ± SEM of three experiments per conditions. (**p < 0.01 vs. control). [file DataSheet1.PDF]

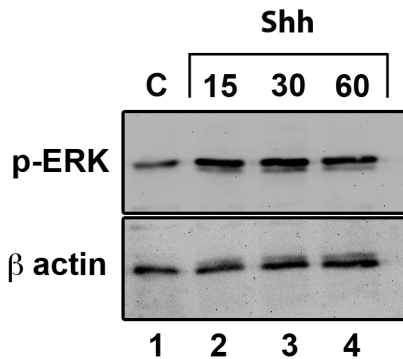

Supplement: Figure S2 — Shh treatment in HeLa cells induces activation of the mitogenic ERK1/2 transduction pathway. HeLa cells treated with 3.3 μg/ml Shh for the indicated time periods showed increased phospho-ERK, detected by immunoblot, maximal at 30 min. [file DataSheet2.PDF]
